# The developing fetal immune landscape and congenital viral infection

**DOI:** 10.3389/fped.2026.1991600

**Published:** 2026-10-29

**Authors:** Sophia C. Chima, Sidney J. Sun, Tyla Duell, Sasha Friese, Vanshika Sindhu, Kristina Adams Waldorf

**Affiliations:** Department of Obstetrics and Gynecology, University of Washington School of Medicine, Seattle, WA, United States

**Keywords:** development, fetus, immune system, ontogeny, pregnancy, teratogen, virus

## Abstract

Fetal development is a complex process that can lead to adverse health consequences when impaired, such as congenital anomalies, fetal growth restriction, preterm birth, and stillbirth. Although there is a well-developed literature on the timing of adaptive immune cell appearance in the fetus, comparatively little is known about the ontogeny and functional maturation of the innate immune system over gestation. This knowledge gap limits our understanding of how intrauterine infections influence fetal development and contribute to adverse obstetric and neonatal outcomes. Thus, the goal of this review is to summarize existing knowledge of the ontogeny of the fetal adaptive and innate immune systems throughout gestation, and how they are impacted by infection. We examine the emergence of hematopoietic stem and progenitor cells, while highlighting changes in immune lineages across the fetal brain, liver, thymus, bone marrow, spleen, and placenta, which play critical roles in immune competency and tolerance. The role of innate immunity in immunocompetence and pathogen responses is highlighted when known. Finally, we address how vertically transmitted and teratogenic viral infections disrupt normal immune and tissue development. Collectively, these studies demonstrate the dynamic, tissue-specific immune landscape that evolves throughout gestation.

## 1 Introduction

For most of the twentieth century, the fetus was considered a passive, immunologically null bystander, incapable of mounting its own immune response. Sir Peter Medawar's characterization of the fetus as a foreign graft, genetically and immunologically distinct from the mother, became the foundation for early thinking in reproductive immunology (1). As a result, the fetus was long regarded as immunologically dependent on maternal mechanisms of immune tolerance for survival and on transplacental transfer of maternal antibodies for immune protection. This theory began to shift around the 1980s, when researchers first provided direct evidence of functional lymphocyte development in fetal tissue. Identification of developing T and pre-B/B cells in fetal liver, bone marrow, and thymus as early as 8–14 weeks of gestation challenged the paradigm of the immunologically passive fetus (2–5). Subsequent research demonstrating antigen-specific fetal immune responses and the capacity for immunological tolerance further established that the fetal immune system was not merely developing structurally, but was functionally active *in utero* (6). More recent work supports the idea that the fetal immune system is not only functional, but that its development is complex and follows a sequence across specific organs. What was once considered quiescent is now understood to actively shape fetal health and disease, with both adaptive and innate immune mechanisms contributing to development long before birth.

Among these mechanisms, adaptive immunity has been the most extensively characterized, largely through studies of umbilical cord blood sampled at birth, with a focus on B- and T-cell function (7). Adaptive immunity refers to the antigen-specific responses mediated by B- and T-lymphocytes, which generate targeted immune memory following pathogen exposure. Fetal adaptive immunity emerges early in gestation and is tightly regulated to prevent inflammation during crucial windows of organ development (8). This developmental bias favors tolerance and tissue protection over pathogen clearance, resulting in reduced inflammatory effector functions and limited use of antigen-specific responses (8). Knowledge regarding innate immune emergence and maturation within the fetal blood and vital organs is more limited. Despite this gap, it is clear that the fetal innate immune system serves several functions during development that extend well beyond host defense. Innate immunity refers to the body's rapid, non-specific first line of immune defense, mediated by cells and mechanisms present from birth rather than acquired through prior antigen exposure. Innate immune cells, such as fetal macrophages, including microglia, participate directly in organogenesis and have been shown to shape the developing brain (9). Fetal innate immunity also matures over time through exposure to potentially immunostimulatory compounds, such as semi-allogeneic antigens from maternal cells, substances from ingested amniotic fluid, and microbial metabolites (10–12). Innate immune cells provide first-line defense both *in utero*, when pathogens breach the placental barrier and during delivery, when the neonate encounters microbes (13, 14). Fetal immune cells have tissue-specific developmental kinetics across organs, providing early cytotoxic and cytokine-mediated surveillance before adaptive immunity matures, while also contributing to organogenesis and host defense development (15).

This review outlines the ontogeny of the fetal immune system across gestation, tracing key developmental events in the fetal liver, brain, spleen, thymus, bone marrow, and placental tissue, before examining the detrimental consequences of *in utero* immune activation in the context of congenital infections and syndromes. As fetal immune development is inseparable from the formation of the organs in which it occurs, understanding fetal organogenesis provides an essential context for interpreting when and where these immune events take place. The following section, therefore, begins with an overview of fetal organogenesis, establishing the structural and temporal framework for the organ-specific immune development discussed throughout this review.

## 2 Fetal organogenesis

Much of our early understanding of organogenesis comes from animal models, but recent studies have made it possible to extend and confirm these findings in a human context. In humans, pregnancy spans approximately 280 days from the last menstrual period to birth (16). It begins with fertilization, followed by cell division and implantation in the endometrial lining. After implantation, the embryonic stage is marked by rapid differentiation into the ectoderm, mesoderm, and endoderm, the primary germ layers from which organogenesis begins. Some studies date milestones in organogenesis from conception, while others follow the obstetric convention of gestational weeks. We have used the latter throughout, adding two weeks to values given as post-conception weeks.

The placenta begins forming upon implantation and matures sufficiently by 10–12 weeks gestation. It takes over progesterone production from the corpus luteum and assumes the developing functions of gas/nutrient exchange and immunological protection (17–19). During embryogenesis, several organs begin to form, which later serve as key immunological niches. Around weeks 4–5, the liver begins to form as an outgrowth of the developing gut tube and the spleen forms from a nearby cluster of supportive tissue attached to the stomach and gut (20). Prior to the start of hematopoietic activity in the liver, neural tubes begin to close while microglial precursors from the yolk sac begin colonizing the forebrain around 4–5.5 weeks (21–23). The thymus emerges slightly later, forming from the third pharyngeal pouch between weeks 6–8 (21, 22). Shortly thereafter, the thymus becomes colonized by hematopoietic progenitors that establish the microenvironment required for T-cell development and selection (21–23).

Other tissue niches relevant to immune function also emerge during this period. In the developing lung, branching morphogenesis generates the conducting airways and epithelial structures, which will later house specialized resident immune populations, such as alveolar macrophages (24). Similarly, formation of the gastrointestinal tract establishes the critical mucosal surfaces and associated tissues that will ultimately support specialized immune populations, like early gut-resident macrophages and innate lymphoid cells (ILCs) (25). These cells begin to organize alongside maternally derived bacterial metabolites already detectable in the fetal intestine, which lays the groundwork for the antigen-sampling and tolerogenic functions that will later define gut-associated lymphoid tissues (12, 25). Collectively, the developmental processes that occur in these fetal organs provide the foundation for a healthy immune system, which can influence immune function and disease susceptibility for the fetus.

Immune cell development during organogenesis begins with hematopoiesis, which originates in the yolk sac and the aorta-gonad-mesonephros (AGM) region. The initial, transient wave of hematopoiesis from the yolk sac generates mainly erythroid and myeloid cell types, while a definitive wave of self-renewing, multipotent hematopoietic stem cells (HSCs) emerge from the AGM (26). These HSCs arise when hemogenic endothelium undergoes an endothelial-to-hematopoietic transition (Figure 1) (27). HSCs are essential to generate blood and immune cells through a tightly regulated process of self-renewal and differentiation. During gestation, HSCs subsequently colonize the fetal liver, which acts as the primary hematopoietic niche through the second trimester, seeding immune populations into other fetal organs (Table 1) (28, 29). This responsibility progressively transitions to the bone marrow in the late first and early second trimester (weeks 11–20), where hematopoiesis is maintained throughout post-fetal life (Figure 2) (26, 30). Defects in HSC development, organ seeding, and differentiation can significantly affect fetal development, leading to profound consequences such as immunodeficiency and abnormal organ development (31). Understanding the timing of these hematopoietic and immune events across fetal organs is therefore essential for characterizing both normal fetal development and its disruption by *in utero* infection.

**Figure 1:**
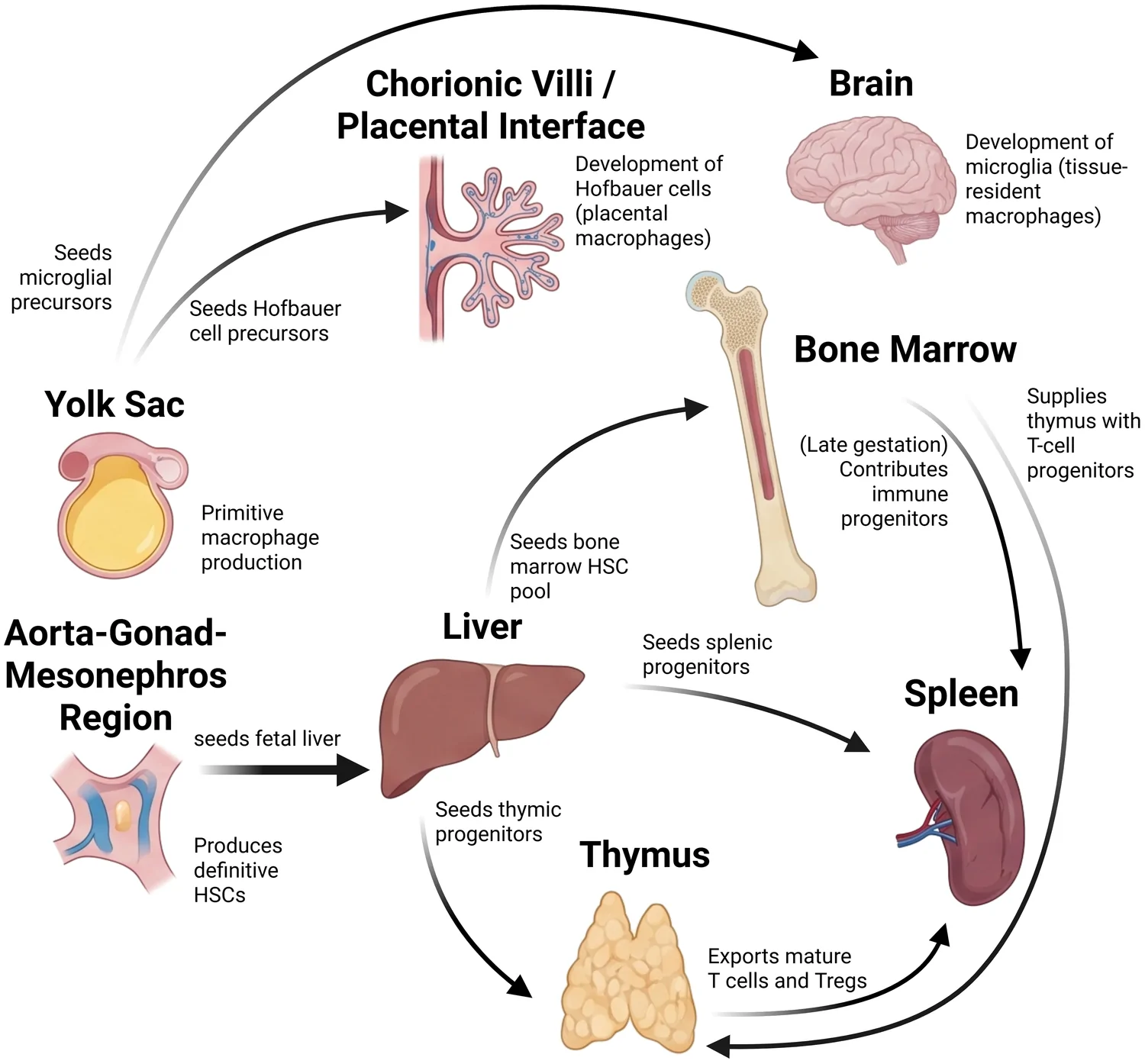
Proposed model of fetal immune cell ontogeny during human development. Yolk sac-derived macrophage progenitors likely seed the fetal brain and chorionic villi, giving rise to microglia and Hofbauer cells. This occurs before definitive HSCs emerge in the AGM and colonize the fetal liver, which serves as the principal hematopoietic organ during early gestation. Hematopoietic progenitors subsequently seed the bone marrow, thymus, and spleen, establishing the immune cell networks that support hematopoiesis and lymphoid development throughout fetal life. The arrows indicate developmental migration pathways between tissues. AGM, aorta-gonad-mesonephros region; HSC, hematopoietic stem cell. Created in BioRender. Chima, S. (2026) https://BioRender.com/tzuu4ax. License: Published under a BioRender individual publication/reproduction license, obtained by the author. Source: BioRender (https://BioRender.com).

**Table 1:** Immune cells and their functions in key human fetal and placental niches.

Organ	Main immune cell types	Gestational age first identified	Primary organ function
Liver	NK cells	6 weeks	Blood cell production and immune development, primary hematopoietic organ through mid-second trimester. Hematopoiesis begins by at least 9 weeks gestation. Seeding hematopoietic lineages into other fetal organs.
	HSCs, erythroid cells, MEMPs, Kupffer cells, myeloid progenitors, megakaryocytes	8 weeks	
	B cells	8 weeks	
	Pre-thymic T cell progenitors	9–10 weeks	
	ILC3	9 weeks	
Spleen	cDC1/cDC2 cells	12–15 weeks	Mediating tolerance to maternal antigens, deficient structural immune function. Blood and immune cell differentiation.
	B cells/progenitors, T cells and Tregs	15 weeks	
	NK cells	15–21 weeks	
	Myeloid progenitors, megakaryote/erythroid progenitors	16–18 weeks	
	ILC-like cells	16–21 weeks	
Thymus	Thymic B1 cells	11 weeks	T-cell maturation and immune selection.
	Thymocytes	10–12 weeks	
	T cells	12–13 weeks	
	Tregs	13 weeks	
Brain	Microglia	4–5.5 weeks	Microglial surveillance, phagocytotic activity, interaction with vasculature in key brain regions during organ development.
	CNS-associated macrophages	5 weeks	
Placenta (chorionic villi)	Hofbauer cells (fetal macrophages)	6–12 weeks	Fetal immune tolerance programming, some antimicrobial defense but can also act as viral transmission site (context-dependent).
Bone Marrow	HSCs	10–12 weeks	Primary hematopoietic organ through late second trimester onward. Expansion of myeloid and lymphoid progenitors in support of immune cell production.
	Diverse multipotent progenitors (myeloid, MEPs, Lymphoid progenitors)	12–14 weeks	
	Granulocytes (including mature neutrophils)	14 weeks	

The table summarizes the timing of immune cell emergence (gestational weeks) and the primary immunological functions of major fetal organs during the first and second trimesters, illustrating the specialization of distinct fetal immune niches. Due to the difficulty in obtaining early fetal tissues, these gestational weeks were often the earliest examined and should not reflect the first emergence. CNS, central nervous system; cDC1, type 1 conventional dendritic cell; cDC2, type 2 conventional dendritic cell; HSC, hematopoietic stem cell; ILC, innate lymphoid cell; MEP, megakaryocyte/erythroid progenitor; MEMP, megakaryocyte-erythroid-mast progenitor; and NK, natural kill cell.

**Figure 2:**
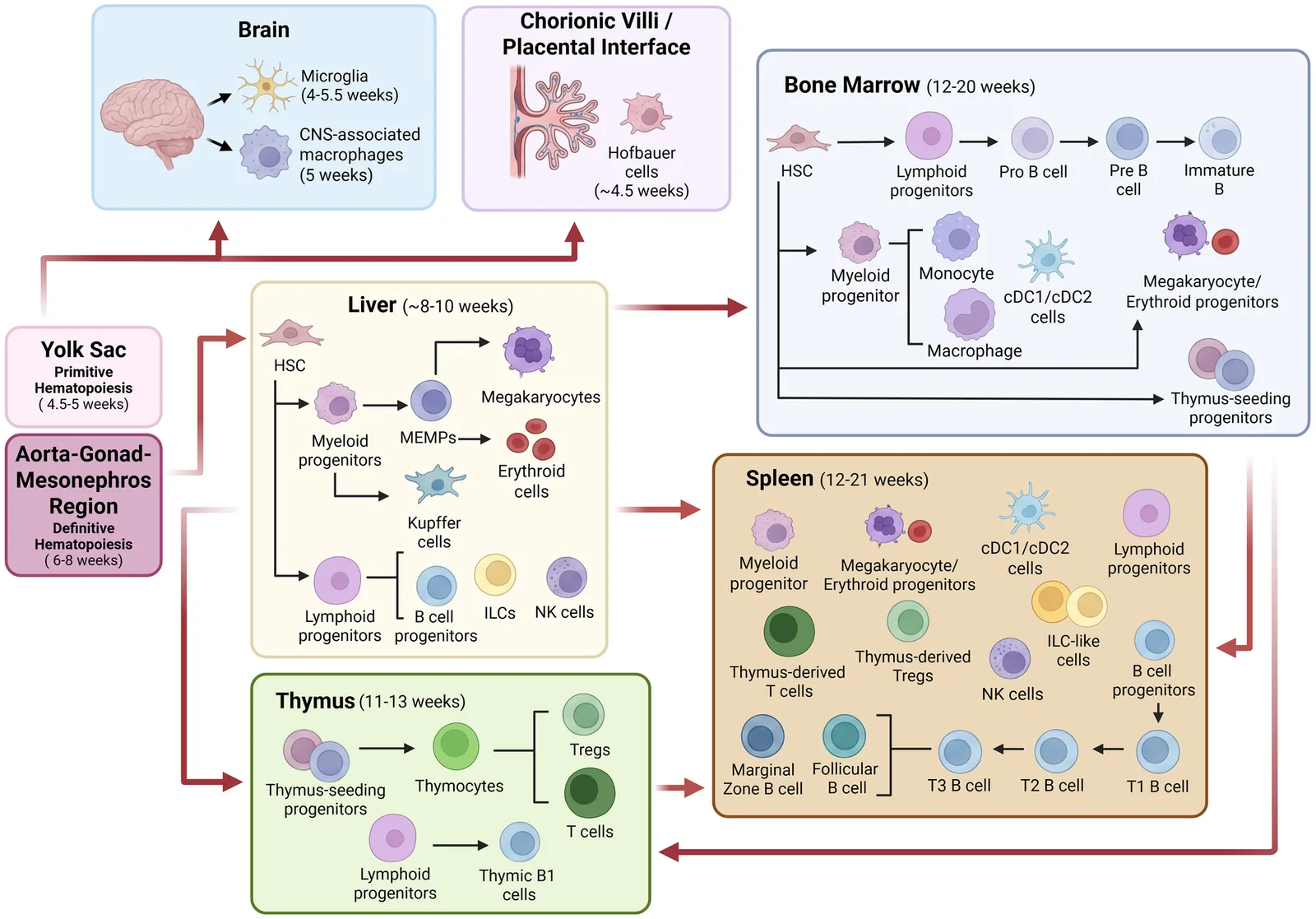
Tissue-specific ontogeny and temporal emergence of immune cell populations across major human fetal organs. This diagram shows the major hematopoietic and immune cell populations detected in each fetal organ niche and the approximate time of first identification noted using the obstetric convention of gestational time from last menstrual period. Detection windows shown for each organ summarize the developmental period during which the illustrated cell populations are first observed. In contrast to the fetal liver, bone marrow, and thymus, which drive hematopoietic differentiation and immune cell development, the function of the fetal spleen is primarily as a secondary lymphoid niche that supports the maturation, maintenance, and organization of developing immune cell populations. Red arrows indicate developmental migration pathways between tissues while black arrows indicate cellular differentiation and lineage relationships. HSC, hematopoietic stem cell. Created in BioRender. Chima, S. (2026) https://BioRender.com/tzuu4ax. License: Published under a BioRender individual publication/reproduction license, obtained by the author. Source: BioRender (https://BioRender.com).

### 2.1 Liver

The fetal liver is the principal hematopoietic organ in early gestation, a function it assumes by 9 weeks gestation, as it supports the early expansion and differentiation of developing immune cells (28, 32, 33). Hematopoiesis in the fetal liver has a unique signature distinct from how adult hematopoiesis is typically understood. The first-trimester fetal liver harbors a CD34 + erythromyeloid-primed multipotent progenitor population, which co-expresses erythroid and myeloid programs (32). Macrophages represent one of the earliest differentiated hematopoietic cell types to populate the fetal liver (34). They appear before the organ's hematopoietic role is fully established and serve initially to promote erythrocyte maturation and the clearing of expelled nuclei (34). In a similar vein, a separate, WNT5A-secreting vascular niche population also supports fetal liver hematopoiesis by sustaining the proliferation of HSCs and progenitor cells. These non-hematopoietic stromal and vascular cells, along with macrophages, actively shape the microenvironment of the fetal liver (35).

The fetal liver plays a central role in seeding developing organs with immune progenitors and supporting early B and T lymphocyte development before these lineages mature elsewhere in the fetus (28, 34, 35). B-cell development emerges early, but its ability to carry out the full range of mature B cell immune functions is highly restricted. Pro-B, pre-B, and surface immunoglobulin-bearing B cells appear in the fetal liver by 8 weeks gestation; by the early second trimester, transitional immature B cells circulate in the fetal bloodstream (3, 36). Although fetal B cells can produce Immunoglobulin M (IgM), Immunoglobulin G (IgG) is derived almost entirely by the mother and is transferred across the placenta in increasing quantities from the second trimester until birth (37, 38). The fetal liver also supports distinct populations of T cells, such as *γδ* T cells, which utilize a restricted receptor gene rearrangement and include an atypical CD4 + CD8− subset not commonly seen among thymic or peripheral *γδ* T cells (39). This subset lacks both the cytotoxic function of CD8+ *γδ* T cells and the classical helper function of CD4+ *αβ* T cells, suggesting it has more of an innate-like surveillance role (39–41). Some T cell progenitors have been shown to be lineage-committed before thymic entry, expressing CD3 complex components and differentiating into mature TCR*αβ*+ T cells when stimulated *in vitro* (42).

Innate immunity also develops early in the fetal liver, where natural killer (NK) cells (mCD3*ε*−/CD56+) and innate lymphoid cells (e.g., ILC3) are among the earliest lymphoid populations detectable in the human fetus, observable as early as 6 and 9 weeks gestation, respectively (Figure 2) (15, 33). Fetal liver NK cells retain their intracellular expression of CD3*γ*, CD3*δ*, and CD3*ε*, which is a T-cell signature, unlike in adult NK subsets (33, 43). This phenotype appears to be developmentally regulated, as CD3*ε* expression during NK cell differentiation can be induced by Notch signaling, a pathway that also governs early T cell commitment (44). Interestingly, this same CD3*ε* expression can re-emerge during the activation of adult NK cells, such as in cases of prior human cytomegalovirus (HCMV) infection (44, 45). Given the fetal liver's essential role as both the principal site of hematopoiesis and early immune development, an *in utero* infection of the liver could have broad consequences for the trajectory of immune competence across various organs.

### 2.2 Thymus

T-cell development begins with lymphoid progenitor cells that seed the thymus around 8–10 weeks post-conception (Figure 1) (21, 46). At this stage, migrating cells express CD34 and CD7, reflecting their progenitor and early T-cell developmental characteristics, respectively. These early thymic progenitors retain multilineage potential and can generate B cells, natural killer cells, and myeloid cells in addition to T cells (47). This occurs prior to progressive lineage restriction to the T cell fate, which becomes complete as they advance through the double-negative stages (47, 48). Immature T cells then progress through the canonical double-negative and double-positive stages, becoming mature by 12–13 weeks (47). These mature T cells can express rearranged T-cell receptors, which enables them to proliferate in response to stimulation (Figure 2). Fetal T cells exhibit distinct gene expression patterns and behaviors from those of adult T cells. Most notably, they exhibit lower levels of interferon (IFN)-*γ* and tumor necrosis factor (TNF), increased interleukin (IL)-10 secretion, and a preference for regulatory T cell (Treg) differentiation compared to adult T cells (49).

The most abundant unconventional fetal thymocytes are Tregs, suggesting that tolerance maintenance is a key function of the thymus (49). Immature and mature gamma-delta (*γδ*) thymocytes are also observed in the second trimester, acquiring effector programs consistent with innate sentinel function (40, 50). Beyond *γδ* T cells, the fetal thymus also contains a distinct population of innate lymphoid cells (ILCs), including a CRTH2⁻CCR9⁺ ILC2 subset that co-expresses T cell-associated genes (51, 52). This suggests that there might be a close developmental relationship between thymic ILC2s and T cell progenitors. Lymphoid tissue inducer (LTi)-like ILC3s are also readily detectable in the fetal thymus and contribute to the formation of functional medullary thymic epithelial cells. These cells are required for T cell selection, indicating that thymic ILCs support the organ's stromal architecture while also representing a distinct lymphoid output (51–53). Compared to mature T cells in peripheral organs, thymic mature T cells show lower expression of TNF-signaling via nuclear factor kappa B (NF-*κ*B) genes and higher expression of IFN-*α* response genes (51, 54, 55). This pattern is consistent with a progressive increase in NF-*κ*B signaling as T cells complete maturation and emigrate from the thymus. Additionally, thymic B1 cells, an innate-like B cell population, persist into the second trimester, even as the B1-to-mature B cell ratio declines in most other organs; this shift likely reflects expansion of mature B cells (51). Collectively, these observations support the presence of a specialized fetal thymic environment that is biased toward immune-regulatory developmental programs.

### 2.3 Bone marrow

The fetal bone marrow becomes colonized by HSCs starting at approximately 13 weeks of gestation, which likely occurs concurrently with the hematopoietic seeding of other secondary lymphoid organs (i.e., spleen) (30, 31). Rather than gradually accumulating lineage diversity over an extended period of time, myeloid and lymphoid cell lineages are established in the bone marrow within a short 6–7 week window following colonization (31). This sets the stage for the fetal bone marrow to gradually replace the fetal liver as the predominant site of hematopoiesis by week 20, which remains the case through adulthood (30). Analysis of the developing bone marrow niche has identified arteriolar endothelial cells and CXCL12-abundant reticular (CAR) cells as the principal sources of HSC-supportive signals, mediating hematopoietic maintenance via NOTCH1/2, CD44, and CD74, while also regulating HSC migration and retention via CXCL12 and related factors (30).

Within this rapid window of lineage establishment, granulocytes, including mature neutrophils, are detectable in the bone marrow by 14 weeks gestation (56). At this stage, progenitors capable of *in vitro* differentiation into neutrophils are also present in both fetal liver and bone marrow, which is consistent with the transitional period in which myelopoiesis occurs in both organs before becoming concentrated in the bone marrow (28, 56). In addition to granulocytes, the bone marrow supports a broad range of other myeloid cell types, including eosinophils and multiple dendritic cell subsets, emerge for the first time during fetal development and are not similarly observed in the fetal liver at the same gestational age (31). Single-cell analyses of second-trimester fetal bone marrow confirm this early diversity, identifying a wide range of hematopoietic progenitors alongside myeloid and lymphoid populations (27). Collectively, these findings illustrate the breadth and complexity of the bone marrow niche as it acquires responsibility for sustaining immune cells (31, 57).

Once the transition to the bone marrow niche is complete, specialized stromal, endothelial, and osteogenic niche populations that organize developing hematopoiesis begin to emerge. These same populations serve as regulators of HSC maintenance and differentiation, helping establish the foundation for lifelong immune cell production (57). Disruption of bone marrow colonization and niche formation by a congenital infection could have long-term consequences for immune function and hematopoietic stability (30, 31, 57).

### 2.4 Spleen

Although the fetal spleen begins to develop around week 5 of gestation, hematopoietic seeding from the liver into the spleen likely takes place around the same time as in the bone marrow and lymph nodes (26). Despite having shared seeding sources, high-dimensional mass cytometry shows that the fetal liver and spleen have different innate immune cell compositions, specifically in progenitor cells and some innate lymphoid cell types (7). Hematopoietic lineages in the human fetal spleen are diverse throughout gestation and highly variable compared with other fetal organs, such as the liver (7). Up until the 14th week of gestation, the fetal spleen is primarily focused on erythropoiesis and myelopoiesis, with a limited population of lymphocytes (20). Around week 15–17, the organ begins to shift towards a more immunological identity. Transcriptomic analysis of the human fetal spleen at 16–18 weeks identifies NK cells, T cells, B cell progenitors, megakaryocyte/erythroid lineage progenitors, and myeloid progenitors as dominating immune populations (Figure 2) (51). Around 16 weeks, the spleen is populated by diverse and mature innate immune cell types, such as ILC2, ILC3, and NK cells (7). The splenic NK subsets are functionally distinct from those found in other lymphoid organs. This is especially true with CD27⁻CD11b⁺ and CD27⁺CD11b⁺ NK cells, which display a dominant signature of immune activation (7). This contrasts with CD27⁻CD11b⁻ NK cells, which are found primarily in the fetal intestine and display a dominant inhibitory signature, showing that immune cell populations in the fetus are shaped by tissue type rather than being uniform across organs. CXCR6⁺ NK cells (58) are also present, and dendritic cells, which are detectable in the spleen by 12 weeks, begin to promote Treg differentiation (10). B cells are yet another notable cell type in the spleen, but at this stage of development they are CD27⁻ and are naïve, likely due to the fact that a memory B cell compartment has yet to emerge (7).

As pregnancy progresses, the fetal spleen develops greater immune competence (20). Weeks 17–26 see the spleen begin to establish the structural foundations required for adaptive immune function later in life, compartmentalizing the incoming T and B lymphocytes in the periarteriolar lymphoid sheath and primary follicle centers, respectively (20). Despite the presence of mature T cells relatively early in the second trimester and their adaptive immune compartments being largely similar to the liver, the immunological tone in the fetal spleen is biased towards tolerance as fetal CD4+ T cells have a strong predisposition to differentiate into tolerogenic Tregs (10, 59). This tolerogenic bias is partly due to the heightened sensitivity of fetal Tregs to TGF-*β*/SMAD and increased expression of Helios (*IKZF2*) (60). The lack of reactive secondary follicles (germinal centers) in the fetal spleen even at term also hinders the activation of an adaptive immune response during gestation, supporting a state of tolerance. However, a recent report built a 23-organ fetal single-cell RNA-Seq atlas and suggested there is greater systemic immune activity than this tolerance model predicts (61). The extent to which systemic immune activation versus a tolerogenic state predominates in the fetus may also depend on microbial and inflammatory exposures.

### 2.5 Brain

During weeks 5–8 of gestation, the central nervous system begins to form and contains a predominance of brain-resident macrophages, called microglia (62). In addition to acting as the primary innate immune defense against pathogens in the fetal brain (63), microglia also support neural differentiation and organization during gestation (9, 64). Specifically, these cells directly shape the number of neurons generated during corticogenesis through selective phagocytosis of progenitor cells, which is distinct from the process used to clear apoptotic debris (9). Microglia have been detected in the human fetal brain as early as 4–5.5 weeks gestation (65–67), which is before hematopoiesis is observed in the fetal liver (9 weeks gestation) (7, 28). Thus, it is probable that the human fetal brain is colonized with immune cells directly from the yolk sac.

Across the different regions of the developing brain, microglia are not uniformly distributed or transcriptionally identical. As early as the second trimester, single-cell analyses have revealed that microglia acquire distinct, region-specific transcriptional identities that become more pronounced across gestation (68). Bulk and single-cell transcriptomic analyses also demonstrate an alternating pattern of microglial proliferation and apoptosis in the human fetal brain throughout development. This is marked by an especially prominent wave of transcriptional activation and microglial proliferation around week 9, indicating the transition from embryonic to fetal identity (65). Microglia mature over the course of gestation, with immunocompetency observed as early as 10 weeks, temporally aligned with peak microglial density in the fetal brain (65). From 9 to 18 weeks, microglial chromatin accessibility (69) and interactions with blood vessels increases (66). Microglial density begins to increase again at week 16, accumulating in white matter tracts between weeks 19–30 before declining after week 35 (70).

The progressive immunocompetence and high density of microglia in the first and early second trimester are accompanied by a consistently activated fetal microglial phenotype (68–70). This suggests that there may be a close relationship between fetal microglia displaying a disease-associated-like transcriptional profile and normal neural development. Although studies of non-microglial immune cells in the human fetal brain are limited, CD8 + and CD4+ T cells, NK cells, and monocytes have been shown to invade the human fetal brain during neuroinflammation caused by infection such as HCMV (71) and Zika virus (ZIKV) (72). This invasion during windows of neuroinflammation is consistent with the well-understood association between congenital neurotropic infections and structural brain abnormalities, including microcephaly, ventriculomegaly, cortical malformations, and intracranial calcifications.

### 2.6 Placenta

The placenta is a complex immune organ that protects the fetus through mechanisms that adapt over the course of pregnancy such as specialized placental cells, fetal-specific immune cells, and maternal antibody transfer. Syncytiotrophoblast (STB) and underlying cytotrophoblast (CTB) cell layers line the villi and directly contribute to maternal-fetal immune tolerance and defense. CTB and STB contribute to maternal-fetal tolerance largely through a restricted human leukocyte antigen (HLA) expression profile. Extravillous CTB express the non-classical, minimally polymorphic HLA-G molecule, while the overlying STB secretes a soluble form, HLA-G5, into the local microenvironment (73, 74). HLA-G engages inhibitory receptors on maternal decidual NK (dNK) cells, macrophages, and T cells, which dampens cytotoxic activity and supports tolerance (74). Although maternal dNK cells are less cytotoxic than peripheral NK cells, they secrete angiogenic and chemotactic factors that promote extravillous trophoblast invasion and spiral artery remodeling (6, 75). However, dNK cells do retain the capacity to acquire cytotoxic function upon activation and have been shown to clear HCMV-infected decidual stromal cells (76). This indicates that the maternal immune compartment of the placenta can shift from a supportive to a defensive state when challenged by pathogens. Additionally, neither STB nor CTB expresses classical HLA class Ia or class II molecules, which limits the antigenic visibility of the fetus to the maternal immune system (73). However, STB can express minor histocompatibility antigens, which can elicit maternal T and B cell responses. These antigens are also present in trophoblast debris, which is continuously shed into the maternal circulation, where it provides a tolerogenic signal to dendritic cells and helps tolerize the maternal immune system to fetal alloantigens (77, 78).

Beyond a tolerogenic HLA profile, the STB also mounts an active antiviral defense in the placenta. Interferon-lambda (IFN-*λ*) is a type III IFN and an important antiviral mediator at the maternal-fetal interface that helps limit transplacental transmission of Zika virus through signaling in maternal tissues (14, 79). Primary human trophoblasts, like STB, constitutively secrete IFN-*λ*1, which protects both trophoblasts and non-placental cells from infection without requiring prior pathogen exposure (14). However, this protection may not be uniform across all placental cell subsets or equally potent across interferon classes. IFN-*λ*1 induces a less robust antiviral gene response than IFN-α in placental macrophages, demonstrating how the response to infection can vary by cell type (80). IFN-*λ* activity is highly gestational stage dependent and provides antiviral protection later in pregnancy. It also potentially contributes to adverse fetal outcomes when activated earlier in gestation (79).

Although STB and CTB govern tolerance and antiviral defense at the surface of the chorionic villi, there is also a limited fetal macrophage repertoire in the stromal core within the villus. These macrophages are known as Hofbauer cells (HBCs) and can be detected in the chorionic villi as early as week 4–4.5 gestational age (80, 81). HBCs have a similar transcriptional signature to primitive yolk sac macrophages (82). In the first trimester, HBCs can display a mixture of both pro- and anti-inflammatory phenotypes. They express innate immune receptors and cytokines to promote placental vascular development, tissue remodeling, maternal-sensing, and pathogen defense (80). However, this activated phenotype changes throughout gestation, with HBCs shifting towards an M2-like tolerant profile closer to parturition (80, 83). This shift is marked by a significant decline in M2a-polarized HBCs and a rise in M2c-polarized HBCs between early and late gestation (80). As previously mentioned, HBCs are the only fetal immune cells found in the villous stroma, and as a result, they are strategically positioned to influence vertical transmission at the maternal-fetal interface. Following pathogen invasion, HBCs can restrict transmission through phagocytosis, cytokine secretion, and other antimicrobial mechanisms. However, several pathogens can infect HBCs, allowing them to act as cellular reservoirs that may facilitate dispersal within the placenta (82). The evolution of HBC tolerance throughout gestation further complicates their pathogen-dependent response to vertical infections from mother to fetus, in some cases promoting viral replication while in other cases playing a defensive role (82, 84–86).

As pregnancy progresses, the immune environment in the chorionic villi shifts towards maternal dominance, with third trimester chorionic villi harboring fewer HBCs and higher proportions of maternal monocytes and macrophages (PAMM1b, PAMM1a, PAMM2, MAC_1) compared to the first trimester (87). The transition to an increased maternal immune-cell presence may help with fetal protection by balancing the inflammatory signaling required for placental maturation and the initiation of birth (88). The shifting immune environment in the chorionic villi throughout gestation, along with the nuanced and pathogen-dependent role of HBCs in vertical transmission, underpins the importance of understanding the impact of gestational age on fetal infection.

Beyond cellular mechanisms of tolerance and defense, the placenta also utilizes a unique, antibody-based transport system to protect the fetus. Maternal IgG is a crucial component of maternal protective immunity that is transferred across the placenta via the neonatal Fc receptor (FcRn) (89). This receptor is expressed on STB cells, allowing IgG to cross the placenta and confer passive humoral immunity (89). The efficiency of IgG is dependent on subclass, gestational age, and antigen specificity. However, the placenta's integrity can be compromised, as some teratogenic pathogens have been shown to use FcRn-mediated mechanisms to cross the placental barrier (90). The transfer of maternal antibodies across the placenta increases after approximately 30–32 gestational weeks, with fetal IgG concentrations rising markedly thereafter (91). Pertussis-specific IgG in the neonate has a half-life of approximately 30 days (92). Maternally transferred IgG ultimately contributes to the broader protection of the fetus by reducing the need for fetal B-cell activation and further limiting the ability to build intrauterine adaptive responses (93).

## 3 TORCH pathogens and congenital viral infections

Having discussed fetal ontogeny and immune cell development in key fetal organs, we now examine the consequences of congenital viral infections for fetal development and health. TORCH is an acronym used to describe a group of pathogens that cause congenital infections and stands for *Toxoplasma gondii* (T), other pathogens (O), Rubella virus (R, RUBV), Cytomegalovirus (C, HCMV), and Herpes Simplex Virus (H, HSV). “Other” TORCH pathogens include human immunodeficiency virus (HIV) and Zika virus (ZIKV), among many others (Table 2) (94–100). Consequences of congenital TORCH infections vary significantly between pathogens, but commonly include severe congenital disabilities such as microcephaly, intrauterine growth restriction, low birth weight, and may even result in fetal death (Figure 3).

**Table 2:** Major TORCH pathogens: gestational risk windows, maternal transmission routes, and available interventions.

Letter	Pathogen (Disease)	Worst Outcomes During Gestation	Transmission Route	Vaccine/Treatment Availability
T	Toxoplasma gondii (Toxoplasmosis)	1st trimester; lower transmission rate but most severe fetal injury	Exposure through undercooked meat or cat feces	No vaccine. Maternal treatment (spiramycin, pyrimethamine-sulfadiazine) may reduce fetal transmission/severity.
O	Treponema pallidum (Syphilis)	Any gestational age; untreated infection can cause fetal loss or severe congenital disease	Primarily sexually acquired infection	No vaccine. Penicillin remains the standard treatment during pregnancy.
O	Zika virus (Zika Fever)	1st trimester (particularly during early neurodevelopment)	Mosquito-borne or sexually acquired infection	No licensed vaccine or specific antiviral treatment. Supportive care only.
O	Varicella-zoster virus (VZV) (Chickenpox)	1st and 2nd trimester (especially 13–20 weeks)	Respiratory droplets or direct contact with infected lesions	Live-attenuated vaccine available before pregnancy. May use maternal varicella-zoster immune globulin (VZIG) and acyclovir following exposure/infection.
R	Rubella virus (Congenital Rubella Syndrome)	1st trimester (especially <12 weeks)	Respiratory droplets	MMR vaccine available and highly effective (pre-pregnancy), but not available globally. No specific antiviral treatment.
C	Human cytomegalovirus (HCMV) (Congenital CMV)	1st and early 2nd trimester; greatest risk for severe neurologic injury	Close contact with infected bodily fluids (i.e., saliva, urine, blood, etc.)	No licensed vaccine or approved maternal antiviral treatment. Antiviral therapy (valganciclovir/ganciclovir) can be used for symptomatic infants.
H	Herpes simplex virus (HSV-1/HSV-2) (Neonatal Herpes)	Greatest risk with late gestation/ peripartum infection	Maternal exposure through sexual contact (HSV-2) or oral contact (HSV-1); peripartum transmission to the neonate	No vaccine. Maternal suppressive acyclovir/valacyclovir and neonatal acyclovir treatment are standard.

This table summarizes major congenital pathogens relevant to fetal development and reviews routes of maternal acquisition, windows of gestational vulnerability, and currently available interventions. As all pathogens listed are capable of vertical transmission, only maternal routes of infection are shown.

**Figure 3:**
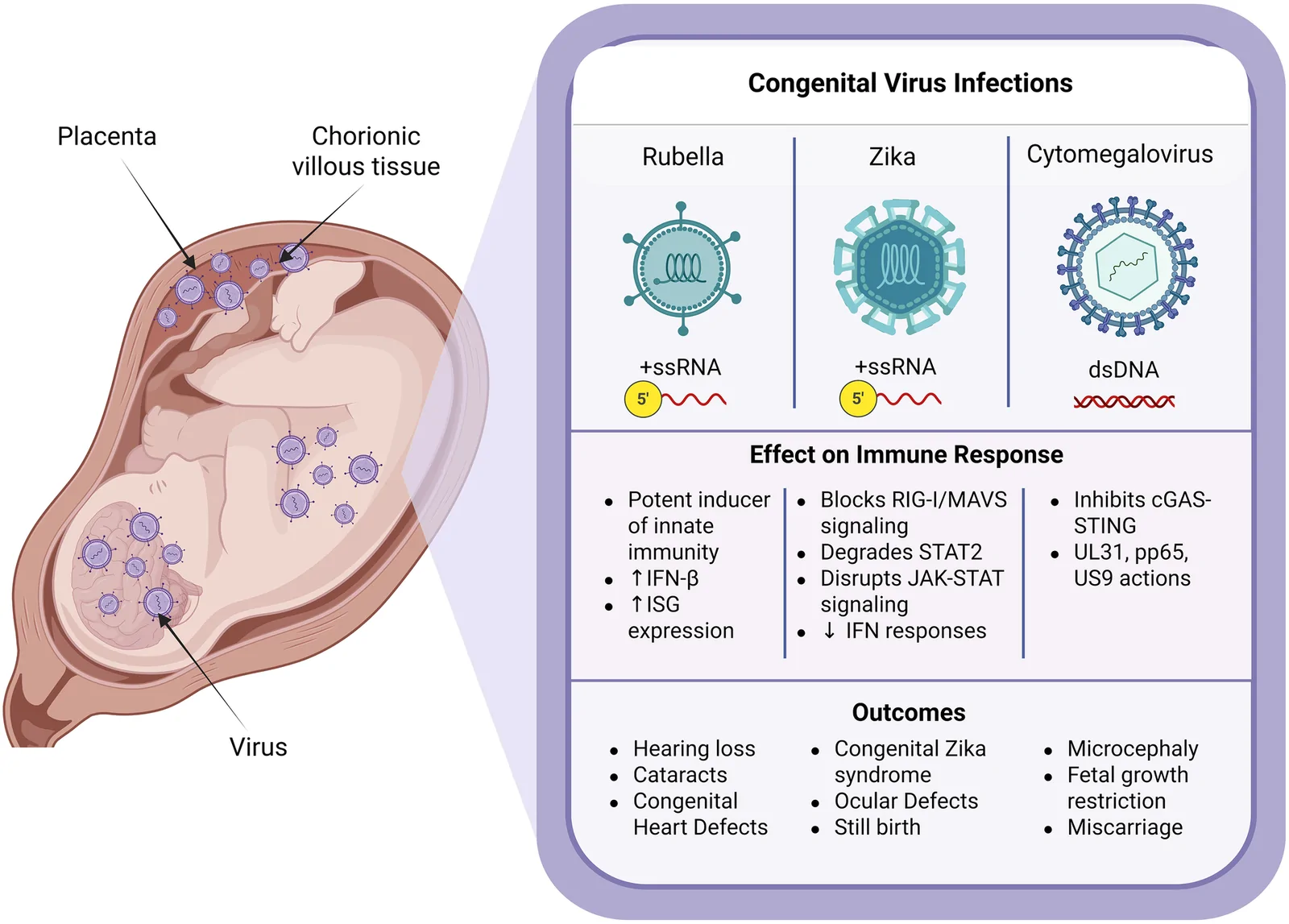
Key characteristics of congenital HCMV, rubella, and Zika virus infection. Schematic comparing characteristics of three major congenital viral pathogens: human Cytomegalovirus (HCMV), Zika Virus, and Rubella Virus. The figure summarizes their congenital outcomes and interactions with the innate immune system. Created in BioRender. Chima, S. (2026) https://BioRender.com/tzuu4ax. License: Published under a BioRender individual publication/reproduction license, obtained by the author. Source: BioRender (https://BioRender.com).

TORCH pathogens are a common cause of infant morbidity and mortality in regions where vaccination and vector control are limited. These infections encompass both vaccine-preventable infections and emerging or endemic threats. For example, cytomegalovirus continues to be the leading infectious cause of congenital anomalies globally, infecting 0.6% of live births (98). Congenital rubella accounted for an estimated 24,000 cases worldwide in 2019 across 19 countries that had not introduced a rubella-containing vaccine into their national childhood immunization schedule (101, 102). In contrast, ZIKV emerged as a major congenital pathogen during the 2015–2016 epidemic in Brazil and has since become endemic in more than 92 countries and territories (103). Though vertical transmission is often considered the most consequential route of infection during pregnancy, congenital viruses can spread to the populace through different transmission routes.

ZIKV is primarily contracted through bites from infected *Aedes aegypti* mosquitoes. However, RUBV spreads through respiratory droplets and HCMV is mostly transmitted through contact with infected body fluids. All three viruses are also capable of being sexually transmitted. These routes of exposure demonstrate the many ways pregnant individuals may encounter congenital pathogens and highlight the importance of population-level prevention efforts. Several clinical and epidemiological studies have shown that both vertical transmission rates and disease outcomes are strongly influenced by the timing of maternal infection. This relationship is evident across multiple TORCH pathogens, which display distinct gestational patterns of transmission and fetal injury. In HCMV infection, maternal infection during the first trimester is associated with the highest risk of severe fetal injury and congenital abnormalities. However, infections later in pregnancy are more likely to be transmitted to the fetus but generally result in less severe clinical outcomes (104–106). Similar patterns have been observed for RUBV, where infection during early pregnancy carries the greatest risk of congenital defects (107). However, unlike HCMV, RUBV transmission rates remain high during both the first and third trimesters but decline during the second trimester (108). For ZIKV, both vertical transmission rates and the risk of fetal injury, including congenital Zika syndrome, decline with each successive trimester (109, 110).

Although the severity of symptoms varies depending on gestational age at infection, ZIKV, RUBV, and HCMV often produce similar fetal outcomes. In adults, infection is frequently asymptomatic or limited to mild symptoms such as fever, rash, and general malaise. One important exception is Guillain-Barré syndrome, which is a rare neurological complication that has been linked to ZIKV infection. Despite a mild symptomatic course in the mother, these pathogens can result in severe adverse outcomes to the fetus such as stillbirth, fetal growth restriction (FGR), and other developmental abnormalities. Congenital ZIKV and HCMV exhibit strong tropism for the developing central nervous system, causing microcephaly, periventricular calcifications, ventriculomegaly, and seizures (99, 111, 112). In contrast, congenital RUBV infection primarily manifests as defects in other areas of the body, such as congenital heart disease, cataracts, hearing loss, and hepatosplenomegaly (96). The discrepancy between mild maternal disease and the severe consequences to the fetus has created a significant clinical challenge. Preventive measures including prenatal screening, mosquito control, and safe sex education can help reduce the risk of maternal infection and its associated adverse fetal outcomes.

## 4 Modulation of host antiviral immune responses

The interaction between TORCH pathogens, activation of fetal immunity, and congenital anomalies is incompletely understood. Interestingly, non-infectious causes of syndromes with a phenotype similar to TORCH infections began to be described in the 1980's. The term “pseudo-TORCH” was first introduced in 1983 to describe brothers with congenital microcephaly and brain calcifications without an infectious cause (113). Studies of pseudo-TORCH syndromes have revealed that persistent activation of type I interferon signaling can produce clinical manifestations that closely resemble those observed following congenital infection. STAT2 gain-of-function mutations and USP18 deficiency, both of which result in excessive type I interferon signaling and uncontrolled ISG expression, are associated with intracranial calcifications, encephalopathy, and other congenital anomalies commonly associated with TORCH-infections (114). An increasing number of genetically heterogeneous disorders have been linked to pseudo-TORCH phenotypes due to dysregulated type I interferon signaling (114–116). Many of these syndromes are part of a broader group of disorders and diseases known as human interferonopathies, which are associated with constitutive activation of type I IFN (117–120). These observations suggest that a strong and prolonged exposure to type I IFN signaling can directly disrupt fetal development and contribute to congenital pathology. This link may explain teratogenesis for some TORCH pathogens.

RUBV, for instance, can elicit strong interferon and ISG responses resulting in various congenital anomalies. Research has shown that infecting adult human neural cells with RUBV robustly induces interferon (IFN)-β at both the mRNA and protein levels (121). Similar findings were observed in organoids derived from mid-gestation human fetal brain tissue, in which neurons and neural progenitor cells mounted strong interferon responses following RUBV infection (122). These findings suggest that RUBV is efficiently recognized by host antiviral sensing pathways, leaving downstream interferon signaling largely intact. Fetal innate immune activation by RUBV likely contributes to the development of the anomalies associated with congenital rubella syndrome.

Other congenital viral pathogens, such as HCMV and ZIKV, are also associated with severe congenital anomalies, but these viruses actively suppress the host innate immune response. With HCMV, the induction of innate immune responses is triggered prior to the expression of any viral gene. Sensing is initiated upon viral attachment and entry, rather than following viral replication (123). A major target of HCMV is the cGAS-STING pathway, a critical sensor of cytoplasmic DNA. Activation of cGAS ultimately leads to production of cGAMP, STING activation, recruitment of TBK1, and downstream induction of type I IFN. After the type I IFN induction pathway peaks around 4–8 h post infection, there is a progressive collapse of the antiviral response due to the accumulation of viral proteins and antagonism of the innate immune response. HCMV interferes with the cGAS-STING pathway at multiple points using the viral proteins, UL31 and pp65 (124, 125). Together, these mechanisms limit pathogen detection and downstream antiviral signaling by inhibiting cGAS enzymatic activity and blocking IRF3 nuclear accumulation. Thus, the virus can suppress antiviral signaling at the level of both pathogen detection and second-messenger generation. HCMV also targets downstream signaling components such as STING and TBK1, preventing efficient propagation of antiviral signals required for interferon induction (126, 127). The breadth of these inhibitory mechanisms highlights the central role of cGAS-STING signaling in controlling HCMV infection. It also illustrates the evolutionary pressure on HCMV to quickly evade immune responses after being sensed by the innate immune system (128).

Similarly, ZIKV shows delayed antagonism of the innate immune response, partly due to the lack of innate immune agonists prepackaged within the virus itself. Host sensors of viral PAMPs induce the type I IFN response, activating JAK-STAT signaling, and expression of interferon-stimulated genes (ISGs). However, over time the viral proteins begin to accumulate, suppressing antiviral immunity at multiple stages of the host response, particularly within the type I IFN pathway. Early on during infection, ZIKV interferes with antiviral sensing through the viral NS4A protein, which disrupts signaling between RIG-I-like receptors (RLRs) and mitochondrial antiviral signaling protein (MAVS) (129). Since the activation of these receptors is a critical upstream pathway for induction of type I IFNs, inhibition at this stage reduces the magnitude of the initial antiviral response and limits downstream ISG expression. ZIKV further disrupts signaling after IFN production has occurred by using the viral NS5 protein and the NS2B-NS3 protease complex. NS5 directly binds and promotes proteasomal degradation of human STAT2, and the ZIKV NS2B-NS3 protease degrades Jak1; together, these actions disrupt signaling downstream of both IFNs and several cytokines (130, 131). This loss of STAT2 and Jak1 signaling impairs transcription of antiviral genes. By targeting both IFN induction and IFN receptor signaling, ZIKV effectively suppresses establishment of the activated, antiviral state, likely contributing to its ability to propagate infection and cause severe neurological injury.

How type I IFN activation and innate immune suppression are balanced in the fetus, and how this balance shapes normal development and the outcome of TORCH infections, remains poorly understood. Pseudo-TORCH syndromes, interferonopathies, and congenital infections all share similar fetal outcomes, yet have vastly different underlying etiologies and temporal activation of the type I IFN response. Even among congenital infections, each virus initiates different mechanisms of the innate antiviral response, including evasion or suppression of type I IFN signaling. In cases of RUBV infection, high levels of type I interferon are produced in the fetus, ultimately leading to congenital abnormalities. On the other hand, during congenital HCMV and ZIKV infection, type I interferon pathway induction is significantly suppressed, but still leads to a similar degree of injury to the fetus. Together, these findings suggest that dysregulated IFN production and signaling can disrupt fetal development. Further investigation is needed to determine how distinct perturbations to the innate immune response converge on similar fetal outcomes.

## 5 Conclusion

Fetal development is a highly dynamic process, and susceptibility to infection changes as fetal organs and the immune system develop. As shown by congenital infections caused by HCMV, Rubella, and Zika virus, both the likelihood of vertical transmission and the severity of fetal injury can vary substantially depending on when infection occurs during pregnancy. These patterns suggest that gestational age influences not only the ability of pathogens to cross the placenta, but also how the developing fetus responds to infection.

Despite increasing interest in fetal immunology, there are still major gaps in our understanding of immune development before birth. Much of what is currently known about fetal immune responses comes from studies of cord blood and neonatal samples. As a result, direct evidence describing adaptive immune responses to infection in the human fetus remains scarce. Similarly, many aspects of fetal innate immune development remain poorly defined. Although the placenta plays a central role in sensing pathogens and innate immune defense during pregnancy, it is unclear how these functions differ among placental cell types, across gestation, or during pathological conditions. In addition, studies of fetal immunity often rely on adult or neonatal immune markers rather than fetal-specific developmental benchmarks. This makes it difficult to determine whether observed differences during infection represent true immune dysfunction or simply normal stages of fetal development.

The current understanding of interactions between congenital viruses and fetal immunity is derived primarily from cell culture studies and mouse models. Comparatively little work has been performed in translational animal models such as nonhuman primates, and even less has focused specifically on fetal immune responses during pregnancy. Although it is well established that disease severity and vertical transmission vary across gestation, the molecular mechanisms underlying these gestational differences remain largely unknown. Addressing these questions will require better experimental models. Ultimately, a better understanding of fetal immune development and placental immunity will be essential for identifying mechanisms of infection-associated fetal injury and developing interventions that reduce the burden of congenital infection, pregnancy loss, preterm birth, and long-term neonatal disease.
